# A central amygdala input to the dorsal vagal complex controls gastric motility in mice under restraint stress

**DOI:** 10.3389/fphys.2023.1074979

**Published:** 2023-02-15

**Authors:** Hao Wang, Wen-Jian Liu, Xi-Yang Wang, Xiao-Qi Chen, Rong-Lin Cai, Meng-Ting Zhang, Hai-Tao Wang, Guang-Wei He, Zhi Zhang, Guo-Ming Shen

**Affiliations:** ^1^ College of Integrated Chinese and Western Medicine (School of Life Sciences), Anhui University of Chinese Medicine, Hefei, Anhui, China; ^2^ Hefei Institute of Pharmaceutical Industry Co., Ltd., Hefei, Anhui, China; ^3^ Department of Thoracic Surgery, The First Affiliated Hospital of Anhui Medical University, Hefei, Anhui, China; ^4^ Research Institute of Acupuncture and Meridian, Anhui University of Chinese Medicine, Hefei, Anhui, China; ^5^ Hefei National Laboratory for Physical Sciences at the Microscale, Division of Life Sciences and Medicine, Department of Biophysics and Neurobiology, University of Science and Technology of China, Hefei, Anhui, China

**Keywords:** central amygdala, dorsal vagal complex, neural circuit, gastric motility disorder, electroacupuncture

## Abstract

**Background/aims:** Psychological and physiological stress can cause gastrointestinal motility disorders. Acupuncture has a benign regulatory effect on gastrointestinal motility. However, the mechanisms underlying these processes remain unclear.

**Methods:** Herein, we established a gastric motility disorder (GMD) model in the context of restraint stress (RS) and irregular feeding. The activity of emotional center—central amygdala (CeA) GABAergic neurons and gastrointestinal center—dorsal vagal complex (DVC) neurons were recorded by electrophysiology. Virus tracing and patch clamp analysis of the anatomical and functional connection between the CeA^GABA^ → dorsal vagal complex pathways were performed. Optogenetics inhibiting or activating CeA^GABA^ neurons or the CeA^GABA^ → dorsal vagal complex pathway were used to detect changes in gastric function.

**Results:** We found that restraint stress induced delayed gastric emptying and decreased gastric motility and food intake. Simultaneously, restraint stress activated CeA GABAergic neurons, inhibiting dorsal vagal complex neurons, with electroacupuncture (EA) reversing this phenomenon. In addition, we identified an inhibitory pathway in which CeA GABAergic neurons project into the dorsal vagal complex. Furthermore, the use of optogenetic approaches inhibited CeA^GABA^ neurons and the CeA^GABA^ → dorsal vagal complex pathway in gastric motility disorder mice, which enhanced gastric movement and gastric emptying, whereas activation of the CeA^GABA^ and CeA^GABA^ → dorsal vagal complex pathway mimicked the symptoms of weakened gastric movement and delayed gastric emptying in naïve mice.

**Conclusion:** Our findings indicate that the CeA^GABA^ → dorsal vagal complex pathway may be involved in regulating gastric dysmotility under restraint stress conditions, and partially reveals the mechanism of electroacupuncture.

## Graphical Abstract

**Figure:**
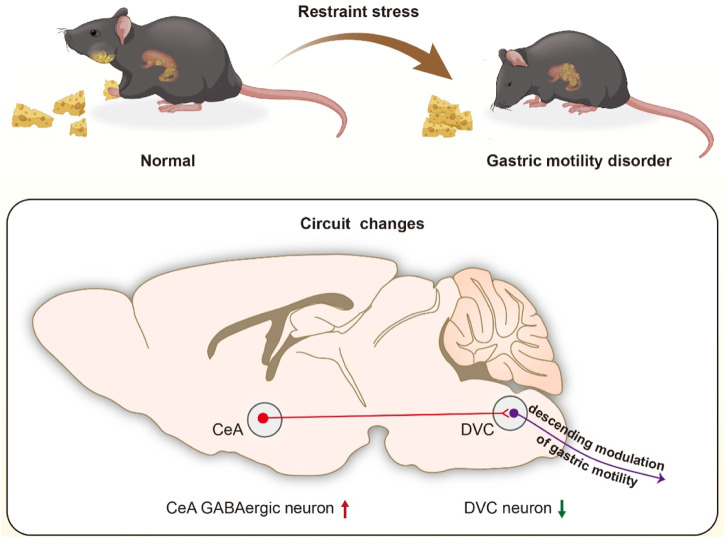
We identified that CeA GABAergic neurons project into the DVC, and proposed that activation of the GABAergic projections from CeA to DVC may be responsible for restrained stress (RS)-induced gastric motility disorder (GMD).

## Introduction

Excessive stress is associated with gastrointestinal motility and mood disorders (Doney et al., 2022). The prevalence of symptoms of depression and anxiety is positively correlated with functional gastrointestinal diseases (FGIDs), and epidemiological data provide evidence that FGIDs and mood disorders interact with each other (Koloski et al., 2020). The high incidence of psychological disorders in FGID patients suggests an intimate and complex link between the gastrointestinal tract and brain, known as the brain-gut axis (Mukhtar et al., 2019; Person and Keefer, 2021). The brain-gut axis suggests how psychological factors directly influence FGIDs by top-down signalling, which is intriguing but poorly understood. The treatment of FGIDs are based on a biopsychosocial model involving the management of physical symptoms and potential psychological comorbidity (Reed et al., 2020; Fikree and Byrne, 2021). Patients with FGIDs frequently also resort to complementary medicine, including acupuncture (Rabitti et al., 2021). Acupuncture is a traditional non-drug therapy that assists the overall regulation of complex systems. In previous studies, we demonstrated that electroacupuncture (EA) at BL21 (Weishu) and RN12 (Zhongwan) promoted gastric movement (Wang et al., 2015). In another clinical functional magnetic resonance imaging (fMRI) project, we demonstrated the effects of acupuncture at RN12 plus BL21 on gastric motility were related to changes in amplitude of low-frequency fluctuations (ALFF) within the amygdala. Moreover, studies have found that EA at the ST36 (Zusanli) improved visceral hypersensitivity and anxiety in functionally dyspepsic rats through inhibition of neuronal discharge of the amygdala (Chen et al., 2022). Therefore, we sought to determine the impact of emotional center, the amygdala, on the modulation of gastric movement upon treatment with EA.

The amygdala is an important component of the limbic system, which is involved in a variety of complex behaviors including emotion, motivation, memory, learning, as well as the modulation of gastric motility (Janak and Tye, 2015; He and Ai, 2016). Mood change is associated with alterated gastric functions (Israelyan et al., 2019). However, the role of this emotional center in gastrointestinal system function remains largely unknown. The amygdala consists of multiple subdivisions, of which the central amygdala (CeA) is the main output nucleus projecting to the brainstem and hypothalamus to control autonomic and motor responses (LeDoux et al., 1988; Sah et al., 2003). Furthermore, physiological and anatomical studies have demonstrated that the CeA projects to the dorsal vagal complex (DVC) which is involved directly in gastrointestinal regulation (Liubashina et al., 2000; Jin et al., 2020). Researchers have uncovered that electrical stimulation of the CeA can alter the basic firing rate of 65% of gastrointestinal-related neurons in the DVC and can also adjust the response of DVC neurons to gastrointestinal stimulation (Zhang et al., 2003). The DVC is composed of the dorsal motor nucleus of the vagus nerve (DMV) and the nucleus of the solitary tract (NTS), and is important for autonomic regulation. Gastric motility is regulated by the vagal pathway originating in the DMV, and the NTS is the recipient of gastrointestinal sensory input (Travagli and Anselmi, 2016). GABAergic neurotransmission to the DMV plays an important role in regulation of gastric motility (Jiang et al., 2019). The CeA is a nucleus predominantly composed of GABAergic inhibitory neurons, and the axonal projections of these GABAergic neurons from the CeA distribute themselves to the midbrain, forebrain and brainstem (Ciocchi et al., 2010; Liu et al., 2021a). Therefore we further investigated the role of CeA GABAergic neurons in targeting and regulation of brainstem DVC neuronal activity to improve RS-induced gastric motility disorders.

## Methods

### Animals

We used Ai9 (RCL-tdT), GAD2-Cre, and C57BL/6J mice (purchased from Jackson Laboratories or Charles River) at 8–10 weeks of age. All mice were maintained at a stable temperature (23°C–25°C) under a 12-h light/dark cycle. Before surgery, mice were housed in cages of six in a colony. The animal protocols were approved by the Animal Care and Use Committee of Anhui University of Chinese Medicine.

### Gastric motility disorder (GMD) model

We used restraint stress (RS) combined with irregular feeding to establish our GMD model. Mice were periodically restrained in a 50 mL syringe for 1 h once per day to constrain their movements and they were fed irregularly (fed on 1 day, fasted for 1 day) for 21 days. Holes were drilled in the syringes to allow the mice to breathe. Control mice were allowed to freely move, and they had *ad libitum* access to food.

### Electroacupuncture (EA) stimulation

**FIGURE 1:**
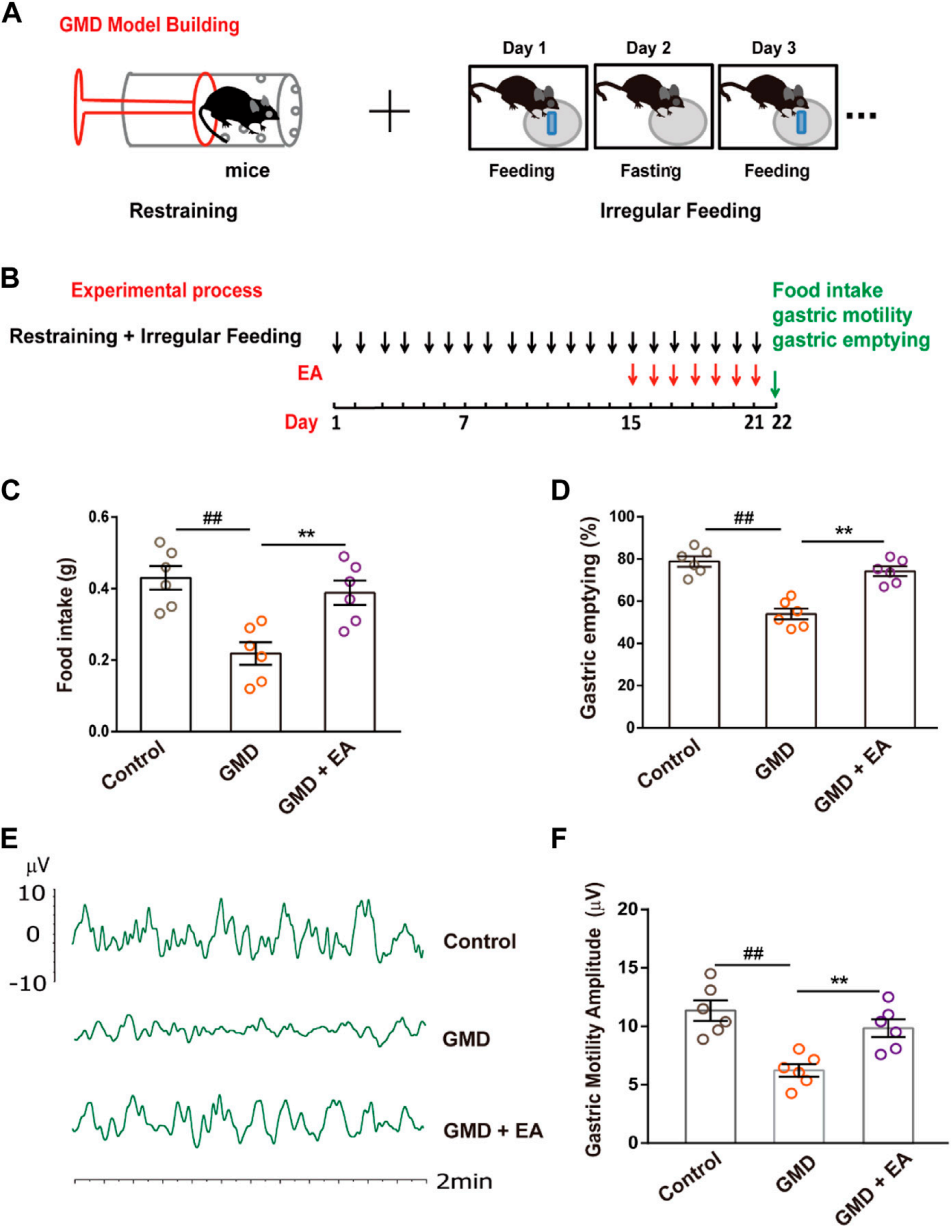
The effect of RS and EA on gastric motility . **(A)** GMD model building; **(B)** An outline of the experimental procedure in GMD mice. **(C–F)** Gastric function tests of food intake, gastric emptying, and gastric motility. (*n* = 6 mice per group). All data are presented as the mean ± SEM. ^##^ *p* < 0.01, ^**^ *p* < 0.01, one-way ANOVA for **(C**, **D, F)**.

The EA group received EA stimulation on the 15th day of the experiment (Figure 1B). Mice were anesthetized by inhalation of isoflurane (1.5%–3.0%) *via* an anesthesia machine. EA was performed with a disperse-dense wave mode for 20 min once per day for 7 days, with electrical current range of 1–2 mA, and a frequency of 2/15 Hz by using a SDZ-IV electronic instrument (Huatuo Brand). EA stimulation was performed at the abdominal RN12 (Zhongwan) and dorsal BL21 (Weishu) acupoints by inserting a 0.18 × 13 mm acupuncture needle. The RN12 acupoint is located on the anterior median line of the upper abdomen, 10 mm below the xiphisternal synchondroses. The BL21 acupoint is located under the spinous process of the 12th thoracic vertebra.

### Measurement of food intake and gastric emptying

Food intake and gastric emptying studies were performed as in other studies (Sampath et al., 2021). Mice were fasted overnight (water available *ad libitum*). The next day, each mouse was caged separately and fed with pre-weighed food for 30 min. In optogenetic experiments, each mouse was fed with light on, and was deprived with light off, the food intake recorded also for 30 min. The remaining unconsumed food was weighed to determine how much food each mouse ate. Food intake (g) = pre-weighed amount-remaining amount. Thereafter, each mouse was placed in a separate clean cage without food and water for 90 min. Mice were then anaesthetized with pentobarbital and euthanized by cervical dislocation. The stomach was removed and weighed (whole stomach weight). The gastric contents were removed from the stomach and the stomach was weighed again (empty stomach weight). The rate of gastric emptying (GE) was calculated as follows: GE(%) = [1—(whole stomach weight-empty stomach weight)/weight of food intake] × 100.

### Measurement of gastric motility

Gastric motility studies were performed as described previously (Wang et al., 2020). Mice were fasted overnight (water *ad libitum*) and anesthetized with pentobarbital (50 mg/kg, intraperitoneally). Following a laparotomy, a miniature strain gauge (1 mm × 1 mm, 120 Ω) was fixed to the circular smooth muscle of the gastric antrum. The laparotomy was then closed with a 5–0 suture with the strain gauge leads exteriorized. Strain gauge signals were amplified, filtered, digitized *via* powerlab 8/30 signal system, and recorded using labchart software (AD Instruments). The waves of gastric contraction were monitored for at least 30 min.

### *In vivo* electrophysiology

A custom-made tetrode array was implanted into the DMV (ML: −0.4 mm, AP: −7.64 mm, DV: −4.45 mm) or CeA (ML: −2.77 mm, AP: −1.22 mm, DV: −4.52 mm) in different batches of mice at the beginning of experiments. The coordinates were defined as dorsal-ventral (DV) from brain surface, anterior-posterior (AP) from bregma and mediolateral (ML) from midline. The screw-based microdrive scaffolds for lowering the electrodes were cemented onto the skull. Each tetrode was made of four twisted fine platinum/iridium wires (diameter 12.5 μm, California Fine Wire). The mice were allowed to recover for 3 days, and the electrodes were attached to a 16-channel signal acquisition system to collect the spontaneous discharge. Then the mice received restraint stress and electroacupuncture treatment. On the next day after the completion of EA treatment, the neuronal activities were recorded, and the data filtered at a bandwidth of 300–5,000 Hz were stored using Neurostudio software. Neuroexplorer 4 (Nex Technologies, United States) was used to calculate the firing rates of the sorted units. We sorted more than 24 units from the recorded spikes of different groups. Only those units with signal noise ratio exceeding 2.5 and average amplitude exceeding 50 μV were included for comparison. However, we removed those with much noise or smaller amplitude to minimize the potential artifact effect.

### Virus injection

We anaesthetized the mice and restrained them in a stereotaxic frame (RWD). A volume of 200 nL of virus was injected into the CeA using a glass microelectrode, which was connected to an infusion pump (micro 4, WPI, United States).

For anterograde tracing of the CeA^GABA^ → DVC circuit, we injected the Cre-dependent virus AAV-DIO-mCherry into the unilateral CeA of GAD2-Cre mice. For inhibition of the CeA^GABA^ neurons or CeA^GABA^ → DVC circuit, we injected the AAV-DIO-eNpHR3.0-EYFP viruses into the bilateral CeA; However, we injected the AAV-DIO-ChR2-mCherry viruses into the unilateral CeA to activate the CeA^GABA^ neurons or CeA^GABA^ → DVC circuit with optogenetic manipulation. The AAV-DIO-mCherry and AAV-DIO-EYFP viruses were used as controls. All viruses were packaged by BrainVTA (Wuhan). 21 days later, the mice were transcardially perfused with 0.9% saline followed by 4% paraformaldehyde. The brain was sectioned for imaging. Images of viral expression were obtained using a confocal microscope (LSM 710; ZEISS, Germany).

### Optogenetic manipulations

An optical fiber (200 µm in diameter) was implanted into the CeA or DVC. The implant was fixed to the skull of mouse using dental cement. The delivery of a 30 min yellow light (594 nm, 5–8 mW, light on 10 min/light off 5 min, three cycles) or 30 min pulsed blue light (473 nm, 2–5 mW, 10 ms pulses, 20 Hz, light on 10 min/light off 5 min, three cycles) was controlled by a master-8 pulse stimulator (A.M.P.I., Israel) (Figures 3D–L). After the experiment, we examined the location of the fibers and removed data from situations in which the fibers weren’t in the proper target.

### Brain slice electrophysiology

#### Brain slice preparation

Anesthetized mice were perfused intracardially with modified ice-cold oxygenated N-methyl-d-glucamine (NMDG) artificial cerebrospinal fluid (ACSF), containing 93 mM NMDG, 1.2 mM NaH_2_PO_4_, 2.5 mM KCl, 20 mM HEPES, 30 mM NaHCO_3_, 2 mM thiourea, 25 mM glucose, 3 mM Na-pyruvate, 5 mM Na-ascorbate, 0.5 mM CaCl_2_, 3 mM glutathione (GSH), and 10 mM MgSO_4_ (osmolarity: 300–310 mOsm/kg, pH: 7.3-7.4). Coronal slices (300 µm) containing the DVC or CeA were obtained using a vibrating microtome (VT1200s, Leica).

Brain slices were incubated in 33°C NMDG ACSF for 12 min and transferred to 25°C N-2-hydroxyethylpiperazine-N-2-ethanesulfonic acid (HEPES) ACSF that contained 92 mM NaCl, 1.2 mM NaH_2_PO_4_, 2.5 mM KCl, 20 mM HEPES, 30 mM NaHCO_3_, 5 mM Na-ascorbate, 25 mM glucose, 2 mM thiourea, 3 mM Na-pyruvate, 2 mM MgSO_4_, 2 mM CaCl_2_, and 3 mM GSH (osmolarity: 300–310 mOsm/kg, pH: 7.3-7.4) for 1 h. Brain sections were put into a slice chamber (Warner Instruments) for recordings, and the slice chamber was continuously perfused with standard ACSF that contained 2.4 mM CaCl_2_, 129 mM NaCl, 3 mM KCl, 1.3 mM MgSO_4_, 3 mM HEPES, 20 NaHCO_3_, 1.2 mM KH_2_PO_4_, and 10 mM glucose (osmolarity: 300–310 mOsm/kg, pH: 7.3-7.4) at ∼3 mL/min.

#### Whole-cell patch-clamp

CeA GABAergic neurons in the slice from *GAD2-tdT* mice were visualized using an upright microscope (BX51WI, Olympus). Current-evoked action potential was recorded in current-clamp mode. Signals were obtained by a Multiclamp 700B amplifier, low-pass filtered at 2.8 kHz, digitized at 10 kHz and analyzed with Clampfit software (Molecular Devices, United States).

#### Light-evoked response

To verify functional characteristics of AAV-DIO-ChR2-mCherry and AAV-DIO-eNpHR3.0-EYFP, pulsed blue light (473 nm, 10 mW, 10 ms pulses) with 5-Hz, 10-Hz, and 20-Hz stimulation protocols, and sustained yellow light (594 nm, 10 mW, 100 ms) were delivered using a laser (Fiblaser, Shanghai) through an optical fiber, respectively. Light-evoked inhibitory postsynaptic currents (IPSCs) were recorded at 0 mV with a blue light (10 mW, 10 ms) in the presence of 4-AP (1 mM) and TTX (1 µM).

### Statistical analysis

SPSS 25.0 software was used for statistical analysis. An unpaired *t*-test was performed for two-group comparisons. ANOVA (two-way or one-way) with a *post hoc* Tukey’s test was used for multiple group comparisons. The significance levels are indicated as **p < 0.01, ##*p* < 0.01. All data are expressed as the mean ± SEM.

## Results

### RS induces gastric motility disorder (GMD), which is alleviated by EA

Physiological and psychological stress can cause FGIDs, and acupuncture improves gastrointestinal motility. To confirm this phenomenon, we established a GMD model in the context of RS and irregular feeding and performed EA (Figures 1A, B). The results illustrated that RS induced delayed gastric emptying, decreased food intake and gastric motility, and EA alleviated GMD (Figures 1C–F).

### RS enhances CeA and suppresses DVC neuronal activity, which is reversed by EA

**FIGURE 2:**
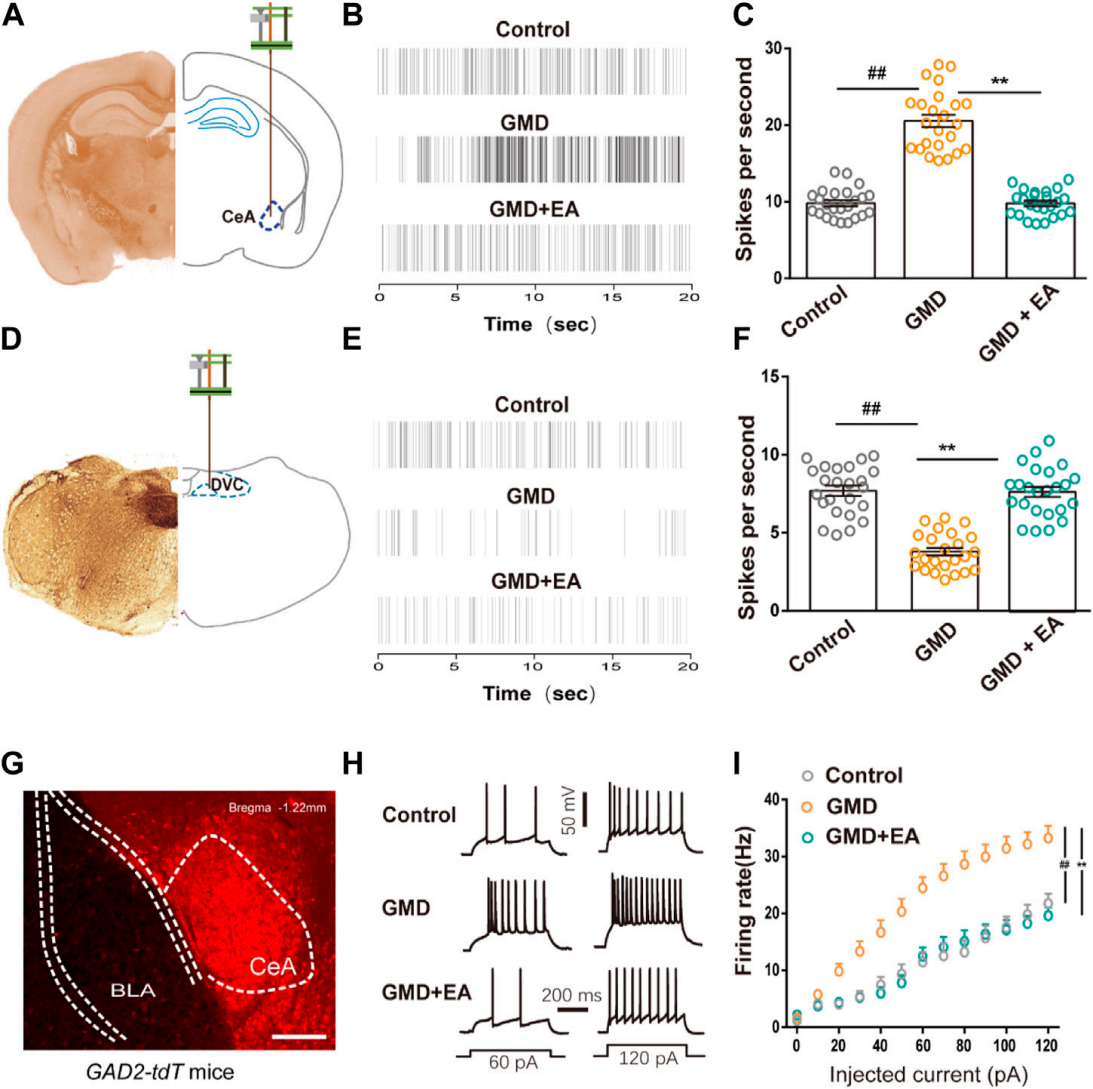
The effect of RS and EA on CeA and DVC neuronal activity **(A, D)** Schematic of *in vivo* electrophysiological recording of CeA and DVC neurons discharge. **(B, C, E, F)** Representative traces **(B, E)** and summarized data **(C, F)** show the firing rate of CeA neurons and DVC neurons in mice with multiple channel recordings (*n* = 24 units from six mice per group). **(G)** Representative image of *GABA-tdTOM* mice (GABAergic neurons with red *tdTOM*). Scale bar, 100 µm. **(H, I)** Sample traces **(H)** and statistical data **(I)** for action potential firings recorded from GABAergic neurons in the CeA in mice (*n* = 18 neurons from six mice per group). All of the data are presented as the mean ± SEM. ^##^ *p* < 0.01, ^**^ *p* < 0.01. One-way ANOVA for **(C, F)**; two-way repeated-measures (RM) ANOVA for **(I)**.

The emotional center (CeA) and gastrointestinal center (DVC) are involved in stress-induced GMD. We recorded the neuronal activity across the CeA and DVC in GMD mice. We demonstrated that RS increased the firing rate of CeA neurons and decreased the firing rate in DVC neurons in free-moving mice, with EA reversing this phenomenon (Figures 2A–F). GABAergic neurons are distributed throughout the CeA, and GABAergic projections play an important role in control of DMV neuronal firing. Therefore, we focused on CeA^GABA^ neurons. Patch clamp recordings were performed in CeA^GABA^ neurons in acute brain slices. To visualize GABAergic neurons, *Ai9* (RCL-tdT) mice were crossed with GAD2-Cre mice to reproduce transgenic mice with red tdTomato-expressing GABAergic neurons (*GAD2-tdT,* Figure 2G). We demonstrated an increase in action potentials with the current-elicited in GMD mice and a decrease in action potentials in GMD + EA mice (Figures 2H, I). These results indicate that CeA^GABA^ neurons and the DVC neurons are involved in the regulation of gastric motility with EA. However, the role of CeA^GABA^ neurons in modulating the activity of DVC neurons to regulate gastric motility is still unclear.

### Inhibition of CeA^GABA^ neurons alleviates GMD

**FIGURE 3:**
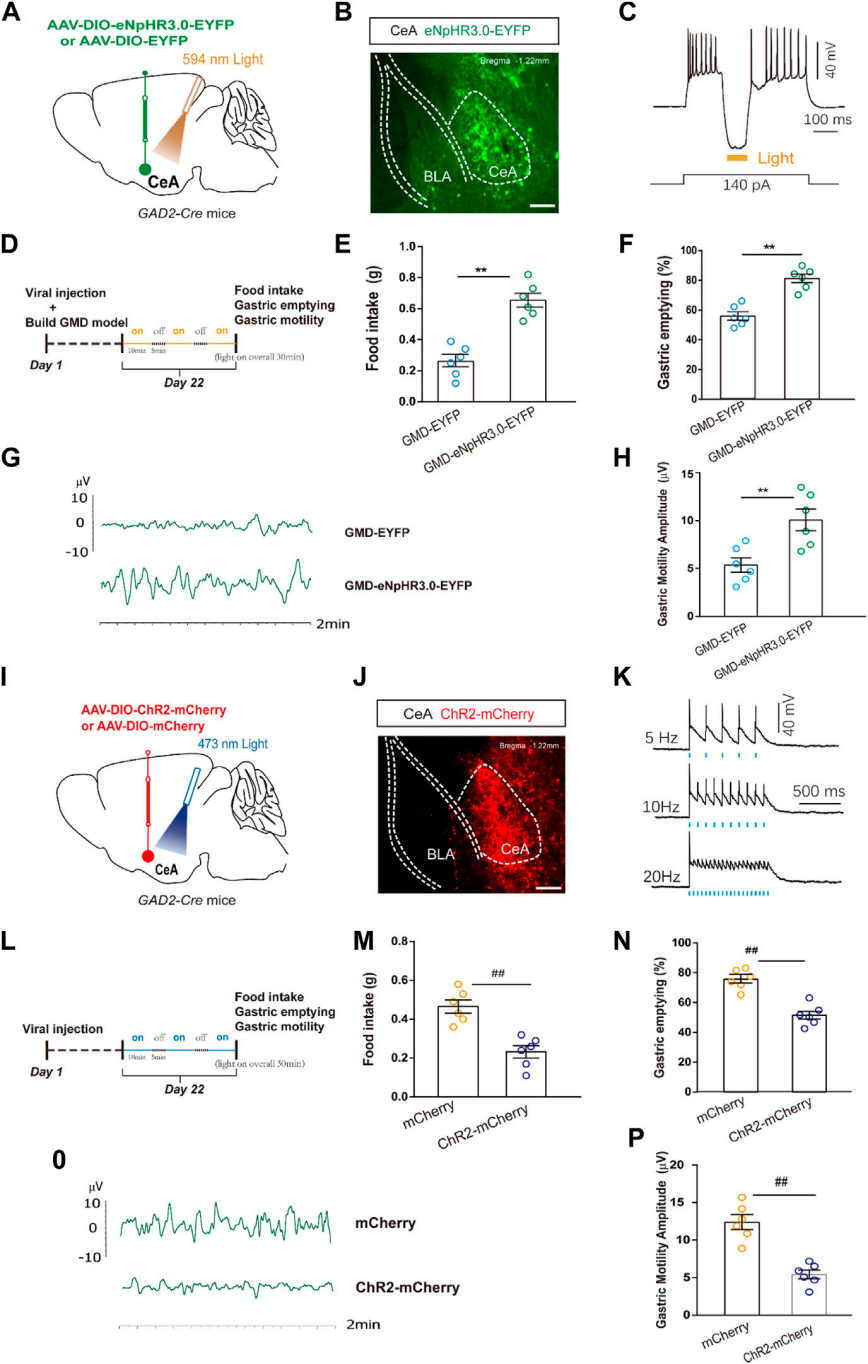
The relationship among CeA^GABA^ neurons and gastric motility. **(A)** Schematic of AAV-DIO-eNpHR3.0-EYFP or AAV-DIO-EYFP viral injection and optic fibre implantation in the CeA. **(B)** Representative image of viral expression in the CeA of *GAD2-Cre* mice. **(D)** Scale bar: 100 μm. **(B)** Sample traces of action potentials evoked by the injected current with photostimulation (594 nm, yellow bar) recorded from eNpHR3.0-EYFP+CeA^GABA^ neurons in acute slices from *GAD2-Cre* mice. An outline of the optogenetic experimental procedure in GMD mice. **(E–H)** Gastric motility effects of the optical silencing of CeA^GABA^ neurons with CeA injection of AAV-DIO-eNpHR3.0-EYFP in *GAD2-Cre* GMD mice [*n* = 6 mice per group, food intake **(E)**, gastric emptying **(F)** and gastric motility **(G, H)**]. **(I)** Schematic of AAV-DIO-ChR2-mCherry or AAV-DIO-mCherry viral injection and optic fibre implantation in the CeA. Representative image of viral expression in the CeA of *GAD2-Cre* mice. Scale bar: 100 µm. **(K)** Sample traces of action potentials evoked by 473 nm light (blue bars) recorded from ChR2 -mCherry ^+^CeA^GABA^ neurons in acute slices from *GAD2-Cre* mice. **(L)** An outline of the optogenetic experimental procedure in naive mice. **(M–P)** Gastric motility effects of the optical activation of CeA^GABA^ neurons [*n* = 6 mice per group, food intake **(M)**, gastric emptying **(N)**, gastric motility **(O, P)**]. All of the data are expressed as the mean ± SEM. ^##^ *p* < 0.01, ^**^ *p* < 0.01. Unpaired *t*-test for **(E, F, H, M, N, P)**.

Given the enhanced CeA^GABA^ neuronal activity in GMD mice, we subsequently aimed to inhibit CeA^GABA^ neurons and observe the change in gastric motility in GMD mice. We injected Cre-dependent eNpHR3.0 into the CeA to selectively suppress CeA^GABA^ neurons. The functionality of the eNpHR3.0 virus was verified by patch clamp (Figures 3A–C). Gastric motility data illustrated that optical inhibition of CeA^GABA^ neurons resulted in significantly increased food intake, gastric movement, and gastric emptying in GMD mice (Figures 3D–H).

In naïve mice, we injected Cre-dependent ChR2 into the CeA to selectively activate CeA^GABA^ neurons. The functionality of the ChR2 virus was verified by patch clamp (Figures 3I–K). The results showed that activation of CeA^GABA^ neurons reduced food intake, delayed gastric emptying and decreased gastric motility (Figures 3L–P). These results establish the functional linkage between CeA^GABA^ neurons and gastric motility.

### An inhibitory pathway from CeA^GABA^ to DVC

**FIGURE 4:**
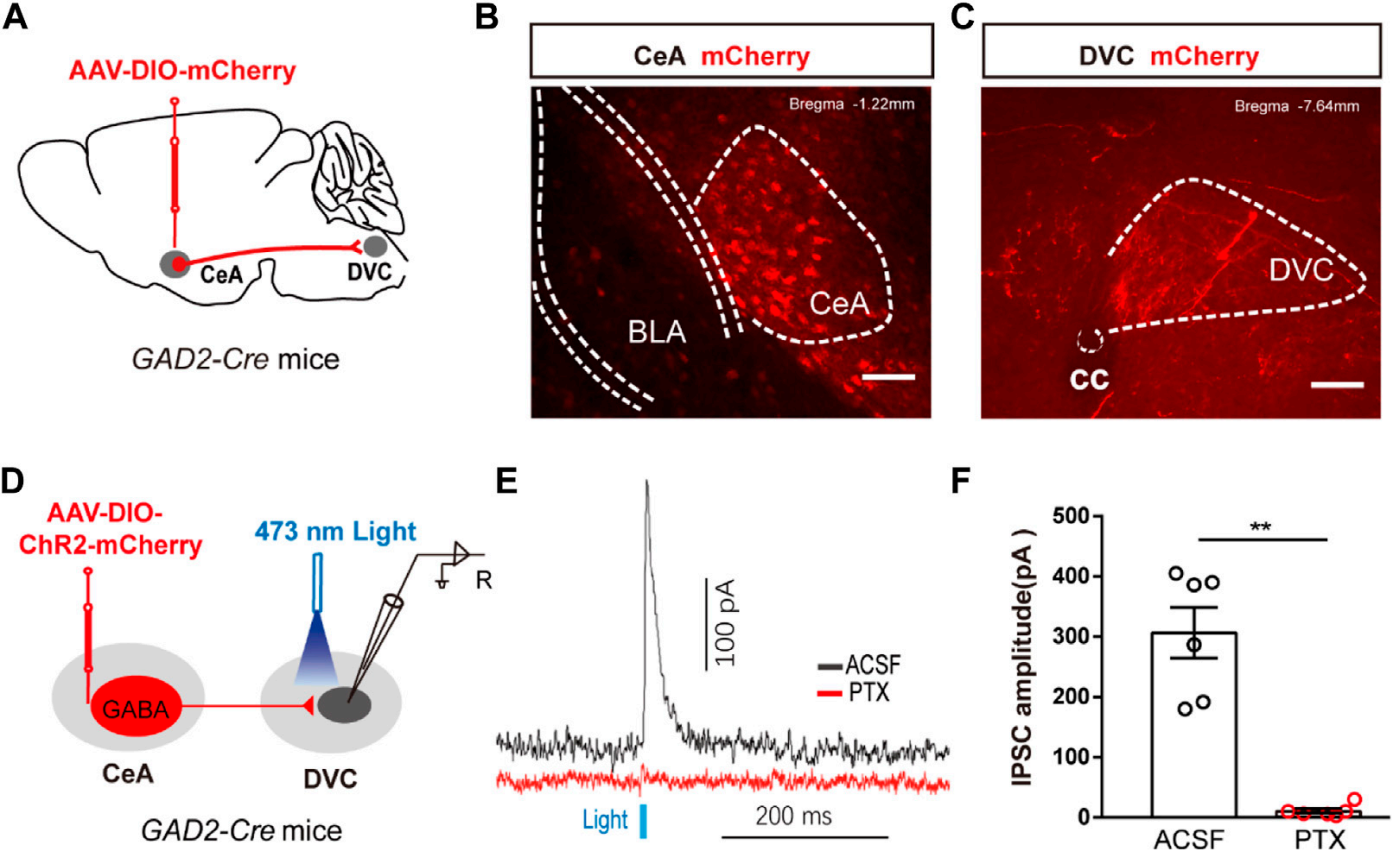
The anatomical and functional connection between CeA^GABA^→DVC pathway **(A)** Schematic of the viral injection. **(B, C)** Representative images of viral expression in the CeA **(B)** and mCherry signals in the DVC **(C)**. Scale bars: 100 µm. **(D)** Schematic of viral injection in *GAD2-Cre* mice and the whole-cell recording configuration in brain slices. **(E, F)** Representative traces **(E)** and summarized data **(F)** of inhibitory postsynaptic currents (IPSCs) in DVC neurons induced by photostimulation (473 nm, blue bars) of CeA^GABA^ terminals in the DVC in the presence of ACSF or the GABA_A_ receptor antagonist PTX (*n* = 6 cells from three mice).

Previous evidence suggested a direct link between the CeA and DVC. To confirm the presence of a CeA^GABA^ → DVC projection, an anterograde transmonosynaptic tracing system was employed. Cre-dependent AAV was injected into the CeA (Figure 4A). 21 days later, we examined mCherry^+^ cell bodies within the CeA (Figure 4B), and numerous additional mCherry^+^ signals were observed in the DVC (Figure 4C). These findings suggest an anatomical connection from CeA^GABA^ neurons to the DVC.

To test the functional connection between the CeA^GABA^ → DVC pathway, brain slice recordings were performed. Brief light stimulation of ChR2-containing CeA^GABA^ terminals in the DVC reliably elicited IPSCs in DVC neurons, which were eliminated by the GABA receptor antagonist picrotoxin (PTX) (Figures 4D–F). These data verify that CeA^GABA^ neurons send inhibitory afferents to the DVC.

### CeA^GABA^ neurons through DVC to regulate gastric motility

**FIGURE 5:**
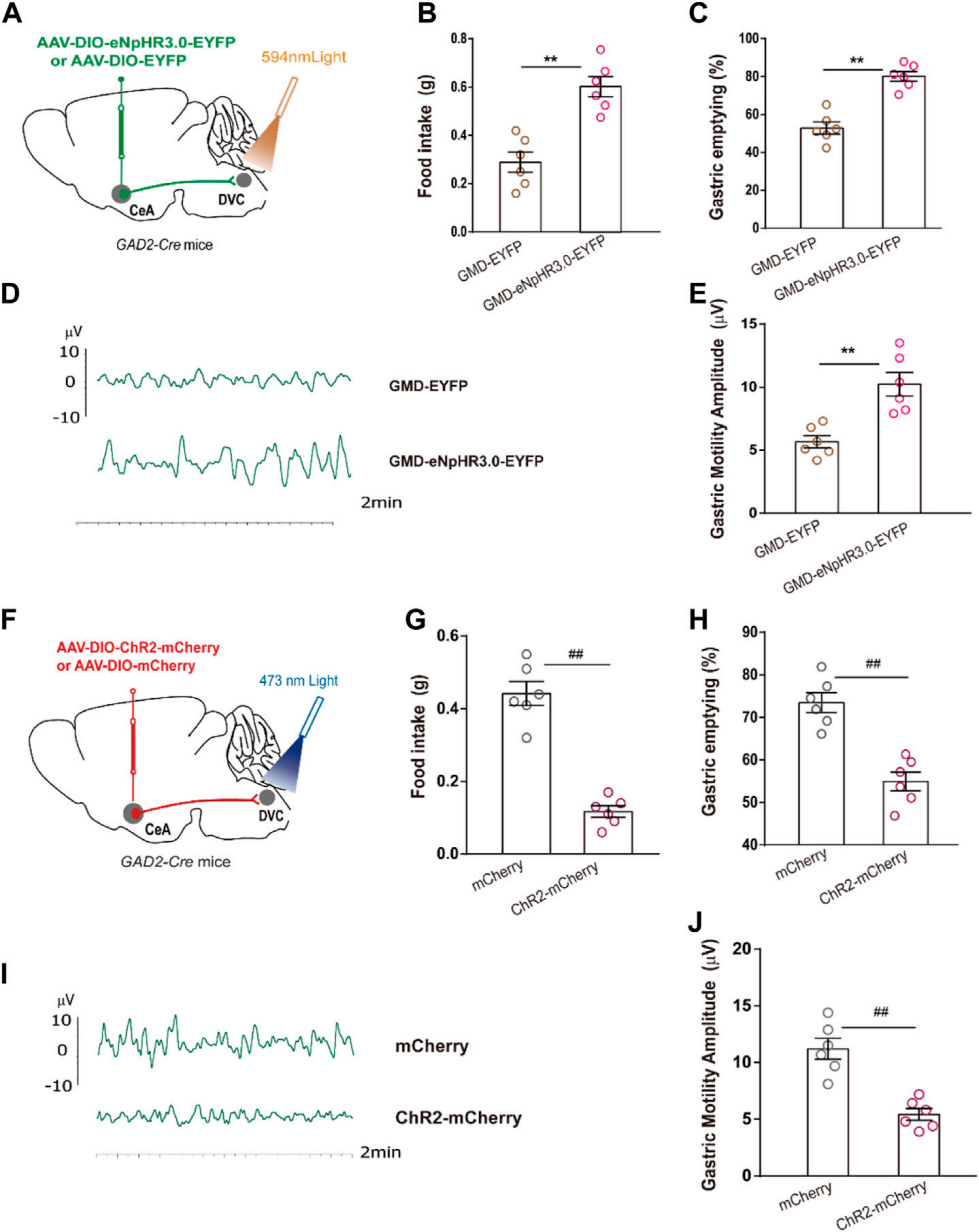
The CeA^GABA^→DVC pathway controls gastric motility **(A)** Schematic of viral injection in the CeA and optical fibre implantation in the DVC. **(B–E)** Gastric motility effects of optical silencing of CeA^GABA^ terminals in the DVC in *GAD2-Cre* GMD mice. [*n* = 6 mice per group, food intake **(B)**, gastric emptying **(C)** and gastric motility **(D, E)**] Schematic of viral injection in the CeA and optical fibre implantation in the DVC. **(G–J)** Gastric motility effects of optical activation of CeA^GABA^ terminals in the DVC of *GAD2-Cre* mice [*n* = 6 mice per group, food intake **(G)**, gastric emptying **(H)**, gastric contraction **(I, J)**]. All of the data are presented as the mean ± SEM. ^##^ *p* < 0.01, ^**^ *p* < 0.01. Unpaired *t*-test for **(B, C, E, G, H, J)**.

We mapped the CeA^GABA^ → DVC pathway to examine whether it participated in regulation of RS-induced GMD. Optogenetic manipulations were employed in GMD mice. We found that optogenetic inhibition of the activity of the CeA^GABA^ → DVC circuit significantly promoted food intake, gastric movement, and gastric emptying in GMD mice (Figures 5A–E). However, in naïve mice, we found that activation of the CeA^GABA^ → DVC circuit reduced food intake, delayed gastric emptying, and subsequently decreased gastric motility (Figures 5F–J). These results suggest that activation of GABAergic projections from CeA to DVC may mimic the symptoms of RS-induced GMD, and inhibition of these projections may relieve symptoms of GMD.

## Discussion

Our study found that activation of the GABAergic projections from CeA to DVC may be responsible for restrained stress (RS)-induced gastric motility disorder (GMD). However, electroacupuncture (EA) is capable of inhibiting CeA and activating DVC neuronal activity to restore gastric motility, partially revealing the mechanism of EA.

Psychological (e.g., fear, anxiety, and anger) and physiological stress (e.g., hunger, overeating, and restraint) can cause FGIDs (Labanski et al., 2020; Chuang et al., 2021; Ge et al., 2022). Many studies have shown that stress delays gastric emptying, inhibits feeding, induces gastric hypersensitivity, and suppresses antral motility in animals (Bulbul et al., 2019; Li et al., 2019; Jiang and Travagli, 2020). Consistent with this notion, we demonstrated that RS and irregular feeding delayed gastric emptying, decreased gastric motility, and food intake.

Chronic stress is a precipitating factor for emotional disorders, which is associated with the effects of chronic stress on the amygdala (Liu et al., 2020a; Song et al., 2020). One study reported that repeated restraint stress increased the neuronal activity of the amygdala (Zhang and Rosenkranz, 2012). Furthermore, another study showed that chronic restraint stress enhanced the activity of CeA GABAergic neurons (Zhu et al., 2019). In our study, we found that the activity of CeA GABAergic neurons was increased in RS-induced GMD mice. However, the functional linkage between CeA^GABA^ neurons and gastric motility remains largely unknown. The connection is confirmed by our findings that optogenetic inhibition of CeA^GABA^ neurons relieved the RS-induced GMD, and optogenetic activation of CeA^GABA^ neurons induced the GMD in naïve mice. Of note, the mechanism of CeA^GABA^ neurons regulation of gastric motility is still unclear.

The CeA is an integrative hub of multisensory information which transforms sensory stimuli of emotional relevance into behavioral and physiological responses, which is accomplished through large efferent fibers sent to a number of downstream nuclei involved in behavioral and autonomic responses (Gilpin et al., 2015). Studies have shown that CeA nesfatin-1 may operate through DMV pathway to regulate gastric distention-sensitive neurons and gastric motility (Wang et al., 2014). Other studies revealed that CeA orexin-A regulated food intake and gastric motility, and that the CeA-DMV-vagus-stomach pathway may be involved in the effect (Jin et al., 2020). Moreover, studies have found that chemogenetic activation of GABAergic neurons from CeA to the lateral hypothalamus (LHA) induced emotional and intestinal motility disorders (He et al., 2022). As the major center of gastrointestinal regulation, the DVC plays an important role in the regulation of gastric motility. Anatomical studies have uncovered that the CeA sends GABAergic projections to the NTS (Saha et al., 2000). In our study, we identified a pathway between CeA^GABA^ → DVC, suggesting that CeA release the neurotransmitter GABA to DVC directly. In addition, injection of GABA-receptor antagonists into the DVC is capable of increasing gastric motility (Sivarao et al., 1998; Babic et al., 2011). Based on our findings, we propose a hypothesis for the neural circuit responsible RS-induced GMD, in which inhibition of the CeA^GABA^ → DVC pathway alleviates GMD, and activation of the pathway results in the symptoms observed in GMD.

Acupuncture stimulation is an ancient practice used to treat human diseases. One core ideal is that stimulation of specific somatic tissues (acupoints) can regulate internal organ function (Liu et al., 2020b). However, the underlying neural mechanism is still poorly understood. Professor Qiufu Ma has proposed that acupuncture effects can be realized through somatosensory-autonomic reflexes, and they found that EA stimulation at ST36 activated the Phox2a projection neurons within the spinal dorsal horn, and the neurons densely project to the NTS (Ma, 2020; Liu et al., 2021b). Furthermore, studies have highlighted acupuncture alter specific brain regions such as cardiovascular regulation-related paraventricular nucleus (PVN) (Cheng et al., 2018), psychomotor response-related CeA (Kim et al., 2021), and drug dependence-related cuneate nucleus (Chang et al., 2019) and so on, which suggest that acupuncture-evoked brain response. Acupuncture stimulation at different acupoints has been reported to drive different autonomic pathways associated with gastrointestinal-motility control (Li et al., 2007). The autonomic nervous system is divided into sympathetic and parasympathetic systems, in which the DMV is the nucleus of origin of parasympathetic nerves. Studies have revealed that EA inhibited the expression of GABA receptors in DMV neurons (Yang et al., 2021). Other studies have found that EA suppressed GABA transmission to DMV to improve gastric motility (Lu et al., 2019). Consistent with previous results, we demonstrated that EA alleviated GMD, inhibition of the GABAergic projection neurons, and activation of DVC neurons in GMD mice. In view of the relationship of CeA^GABA^ → DVC pathway and gastric motility, we proposed that EA regulated gastric motility may through modulate the activity of CeA^GABA^ → DVC pathway. Admittedly, limitations of this study include: 1) Our way of measuring gastric emptying rate did not eliminate the compounding effect of gastric secretion, and ideally the gastric content should have been dried. 2) Our acupuncture research is rather preliminary, although the study provides a new outlook for the research of mechanism of acupuncture from the perspective of neural circuit.

Overall, the CeA sends GABAergic projections to DVC which contribute to restraint stress-induced gastric motility disorder, which provides a neuroanatomical explanation for stress-induced GMD. Moreover, our study also reveals the overall regulatory mechanism effects of acupuncture.

## Data Availability

The original contributions presented in the study are included in the article/supplementary material, further inquiries can be directed to the corresponding author.
